# Paradoxes in approaching the most prevalent infections in ENT practice: tonsillitis, sinusitis, and otitis media

**DOI:** 10.1016/S1808-8694(15)31370-7

**Published:** 2015-10-17

**Authors:** Tania Sih

**Keywords:** tonsillitis, otitis media, sinusitis, anti-bacterial agents

Young children going to day care centers complaining of secretion, nasal obstruction, and cough can be seen on a daily basis in the pediatric ENT practice. The signs and symptoms of viral and bacterial respiratory infections are often juxtaposed to allergy, leading physicians to prescribe less-than-adequate drugs to treat the conditions at hand. Most prescription errors are due to inaccurate diagnosis, as interviews and clinical examination are neglected and test results are misinterpreted.

Until some time ago Medical Doctors used to appreciate the value of a good interview with the patient, properly conducted clinical examination, and the epidemiologic data gathered from the community he or she served. Objective clinical data was abundant, whereas ancillary tests were not amply available. Today, lab tests and imaging technologies that some decades ago were virtually unthinkable are reasonably available, depending on the site and affordability.

On the other hand, this change has led many physicians to develop therapies based on ancillary test reports (often excessively ordered), instead of fitting the pieces of the puzzle taking into account the signs and symptoms (along with physical examination) mentioned by the patient. In other words, physicians are treating reports instead of patients. Thorough interview and properly conducted physical examination are being traded for interpretation of reports and test results – based on often poorly indicated exams thus originating prescription errors.

The opposite also happens, and that may be the case most of the time in developing countries. Physicians working at emergency units that see a large number of children without knowing anything on their history and unable to assess patient clinical evolution within the following 24 or 48 hours, tend to diagnose and treat most acute respiratory infections as bacterial disease. Antibiotics are often prescribed, mainly when the resources needed to enhance bacterial disease diagnostic certainty are not available.

When looking at respiratory diseases, the two situations described above will objectively lead to diagnosis without disease; patient complains of a sore throat, test for oropharyngeal strep infection is not available, patient is prescribed antibiotics for viral infection; hyperemic tympanic membrane (TM) when patient cries, patient is wrongly diagnosed with acute otitis media or even TM opacity due to effusion otitis media and, consequently, is offered antibiotics; diagnosis of sinusitis using a simple x-ray image done as the child cries or in the early stages of a cold, with poor visualization of the paranasal cavity, leading to unnecessary prescription of antibiotics. This is a true radiologic sinusitis epidemic. All situations above induce medication abuse.

Patient interview and careful physical examination and, in some circumstances, ancillary tests, are fundamental to produce accurate diagnosis. Physicians also need to get more acquainted with the principles of evidence-based care in order to select the most adequate therapy for each patient, bearing in mind that the efficacy of many commonly prescribed drugs is not proven as they may produce severe adverse effects.

In the upper airways, the three diseases most commonly associated with overmedication errors are tonsillitis, sinusitis, and acute otitis media.

It is important, therefore, that PATIENT INTERVIEW and DIAGNOSIS (based on complementary tests) are firmly connected to each other, so patients are offered the proper treatment based on evidence-based medical care.

